# Prevalence and Factors Associated with Burnout Syndrome among Primary Health Care Nursing Professionals: A Cross-Sectional Study †

**DOI:** 10.3390/ijerph17020474

**Published:** 2020-01-11

**Authors:** Magno Conceição das Merces, Julita Maria Freitas Coelho, Iracema Lua, Douglas de Souza e Silva, Antonio Marcos Tosoli Gomes, Alacoque Lorenzini Erdmann, Denize Cristina de Oliveira, Sueli Bonfim Lago, Amália Ivine Costa Santana, Dandara Almeida Reis da Silva, Maria Lúcia Silva Servo, Carlito Lopes Nascimento Sobrinho, Sergio Corrêa Marques, Virgínia Paiva Figueiredo, Ellen Marcia Peres, Marcio Costa de Souza, Luiz Carlos Moraes França, Deborah Monize Carmo Maciel, Álvaro Rafael Santana Peixoto, Pablo Luiz Santos Couto, Marília de Souza Maia, Márcia Cristina Graça Marinho, Silvana Lima Guimarães França, Claudia Franco Guimarães, Klaus Araujo Santos, Fábio Lisboa Barreto, Janaína de Oliveira Castro, Milene Pereira de Souza Santos, Milena Oliveira Coutinho, Kleyton Góes Passos, Roberto Rodrigues Bandeira Tosta Maciel, Fernanda Warken Rosa Camelier, Argemiro D’Oliveira Júnior

**Affiliations:** 1 Department of Life Sciences, State University of Bahia (UNEB), Salvador 41150-000, Bahia, Brazil; julitamaria@gmail.com (J.M.F.C.); sbonfimlago@gmail.com (S.B.L.); daraareis@gmail.com (D.A.R.d.S.); mcsouza@uneb.br (M.C.d.S.); deborahmonizecm@gmail.com (D.M.C.M.); marinho_m@hotmail.com (M.C.G.M.); sillgf@hotmail.com (S.L.G.F.); kau.araujo25@gmail.com (K.A.S.); janaocastro@outlook.com (J.d.O.C.); profmilenescmed@gmail.com (M.P.d.S.S.); mile-rosa@hotmail.com (M.O.C.); robertorbtm@hotmail.com (R.R.B.T.M.); fwrcamelier@gmail.com (F.W.R.C.); 2 Department of Health, State University of Feira de Santana (UEFS), Feira de Santana 44036-900, Bahia, Brazil; ira_lua@hotmail.com (I.L.); luciaservo@yahoo.com.br (M.L.S.S.); mon.ica@terra.com.br (C.L.N.S.); lisboa.auditor@gmail.com (F.L.B.); 3 Health Sciences Postgraduate Program, School of Medicine, Federal University of Bahia (UFBA), Salvador 40026-010, Bahia, Brazil; douglasss-gbi@hotmail.com (D.d.S.e.S.); amalia0807@gmail.com (A.I.C.S.); mariliasmaia.fisio@gmail.com (M.d.S.M.); franconurse@gmail.com (C.F.G.); argemiro@ufba.br (A.D.J.); 4 School of Nursing, State University of Rio de Janeiro (UERJ), Rio de Janeiro 20551-030, Rio de Janeiro, Brazil; mtosoli@gmail.com (A.M.T.G.); dcouerj@gmail.com (D.C.d.O.); sergiocmarques@uol.com.br (S.C.M.); virginiafigueiredo@yahoo.com.br (V.P.F.); ellenperes@globo.com (E.M.P.); lcmoraesfranca@hotmail.com (L.C.M.F.); 5 Postgraduate Program in Nursing, Federal University of Santa Catarina (UFSC), Florianópolis 88.040-900, Santa Catarina, Brazil; alacoque.erdmann@ufsc.br; 6 Postgraduate Program in Psychology, Center for Human and Natural Sciences, Federal University of Espírito Santo (UFES), Vitória 29075-910, Espírito Santo, Brazil; alvarorafael.peixoto@gmail.com; 7 Department of Education, State University of Bahia (UNEB), Guanambi 46430-000, Bahia, Brazil; pabloluizsc@hotmail.com; 8 Multidisciplinary Center, Federal University of Acre (UFAC), Cruzeiro do Sul 69980-000, Acre, Brazil; kleyton.ufac@gmail.com

**Keywords:** burnout, nursing, Primary Health Care

## Abstract

The objective of the study was to evaluate the prevalence and factors associated with Burnout Syndrome (BS) in Primary Health Care (PHC) nursing professionals from the state of Bahia, Brazil. A multicentre, cross-sectional population-based study was conducted in a cluster sample among 1125 PHC Nursing professionals during the years 2017 and 2018. We used a questionnaire that included sociodemographic, labor and lifestyle variables and the Maslach Burnout Inventory scale to identify BS. The associations were evaluated using a robust Poisson regression with the hierarchical selection of the independent variables. The prevalence of BS was 18.3% and the associated factors were ethnicity (prevalence ratio (PR) = 0.62, confidence interval (CI) 95% = 0.47–0.83), residence (PR = 2.35, CI 95% = 1.79–3.09), economic situation (PR = 1.40, CI 95% = 1.06–1.86), satisfaction with current occupation (PR = 1.75, CI 95% = 1.31–2.33), (PR = 1.60, CI 95% = 1.23–2.08), rest (PR = 1.83, 95% CI = 1.41–2.37), technical resources and equipment (PR = 1.37, CI 95% = 1.06–1.77), night shift (PR = 1.49, CI 95% = 1.14–1.96), physical activity practice (PR = 1.72; CI 95% = 1.28–2.31), smoking (PR = 1.82, CI 95% = 1.35–2.45), and satisfaction with physical form (PR = 1.34, CI 95% = 1.01–179). Strategies are needed to prevent BS, with an emphasis on implementing worker health programs in the context of PHC.

## 1. Introduction

Labour has undergone significant changes throughout history, despite advances in the industrial and technological area. Especially in countries with advanced Capitalism, many transformations linked to those changes have taken place in labour relations, such as the growth of informality, outsourcing and subcontracting, which became commonplace over that time. Thus, economic and industrial development cannot be associated with a direct relationship with the improvement of workers’ conditions, especially in countries with intermediate industrialization, such as Brazil [1,2].

Working conditions not only determine issues related to materiality, but also imply changes on individuals. Precariousness, informality, and the need for triple journeys consume not only workers’ time, but also their way of being; that is, their body and psyche. Technological development and production can be inadvertently associated with the development of the individuality and autonomy of the worker. However, those relations are rather linked to the onset of illness, making work a sacrifice for individuals in their daily life [1,3,4,5].

In this scenario, Burnout Syndrome (BS) stands out and is defined as a state of physical and emotional exhaustion because of long exposure to stressful work environments and unmanaged chronic occupational stress. The three main dimensions of the syndrome are the following: Emotional Exhaustion (EE), Depersonalization (DP)—also known as feelings of cynicism and distancing from work—and Reduced Professional Achievement (RPR)—understood as a feeling of ineffectiveness [6,7]. BS impacts absenteeism, presenteeism, decreased productivity, an increased risk pf occupational accidents, alcohol and psychotropic abuse, time off work, and cardiometabolic diseases, among others [8,9,10].

BS has recently been included in the International Classification of Diseases, 11th Issue (ICD-11) as an occupational phenomenon [11]. Some studies conducted since the 1970s have justified this inclusion and have already indicated a high prevalence of the disease and deleterious effects on health and workers’ absence. Professionals who have a constant and direct relationship with different people are more exposed to BS [12]. This group includes teachers, nursing professionals, psychologists, physicians, social workers, police officers, and fire fighters, among others [12]. Among health professionals, nursing professionals have one of the highest BS rates [13]. There is a greater number of studies among nursing professionals and physicians working in hospitals, especially in intensive care and urgency/emergency units [14,15,16].

However, representative studies that evidence the prevalence and factors associated with BS in Primary Health Care (PHC) nursing professionals are scarce. Survey information regarding the prevalence and risk factors of BS is needed to prevent the syndrome and determine the most appropriate clinical interventions. Therefore, this study aims at evaluating the prevalence and factors associated with Burnout Syndrome (BS) in Primary Health Care (PHC) nursing professionals from the state of Bahia, Brazil.

## 2. Methods

A cross-sectional and exploratory study based on data from a multicentre population-based epidemiological survey was conducted in the state of Bahia, Brazil. Bahia is the fourth most populous Brazilian state, consisting of 417 municipalities, and is organized in seven mesoregions, namely the Western Mesoregion, São Francisco Valley Mesoregion, Central Northern Mesoregion, Northern Mesoregion, Metropolitan Mesoregion of Salvador, Central Southern Mesoregion, and the South Mesoregion. All regions together total 15 million inhabitants [17].

A cluster sampling of PHC nursing professionals was taken from the state of Bahia, Brazil. Only 10% of the municipalities (clusters) were drawn from each stratum (mesoregion), totalling 43 municipalities. Microsoft Office Excel 2010 was used for the evaluation. We included all PHC nursing professionals of the drawn clusters, totalling 1195 individuals.

No robust studies were found in the literature that have explored the BS rate among PHC nursing professionals, which led us to conduct a pilot study in a similar population to obtain parameters for sample calculation. The BS rate sare 20% and 33.3% in the non-exposed and exposed groups, respectively, with an error *α* of 0.05, a power of 90% (1 − error *β*), and a ratio 1:1, reaching a sample *n* of 464. The design effect of 2.0 (sample by clusters) was also considered; the sample was doubled to 928, and we added 20% to the possible losses and refusals. Therefore, 1114 PHC nursing professionals were obtained. The EPI Info 7 software (Center for Disease Control and Prevention, Atlanta, USA) was used to calculate the sample size.

All PHC nursing professionals from the 43 municipalities (1114 individuals) were invited to participate in the study. Individuals with the following statuses were excluded: on sick leave; with less than 6 months of experience in PHC; in solely administrative work routines; pregnant women; women in their menstrual period; and individuals with a diagnosis of depression, anxiety and BS before occupying the current position, as well as liver cirrhosis and alcohol and drug addiction. By applying these criteria, 48 professionals were excluded and 22 refused to participate in the research. The final sample consisted of 1125 participants. The response rate was 94.1%.

Data collection took place in 2017 and 2018, at health units, in a private office, through anamnesis—a psychometric scale and a questionnaire that includes variables related to sociodemographic, occupational and lifestyle data. To ensure homogeneity in the application of the scale and the questionnaire, a calibration was performed among the research assistants by interviewing 30 hospital professionals. The agreement among the assistants was calculated using the Kappa index, which was 0.87 and considered acceptable [18].

The data collection instrument was the Maslach Burnout Inventory—Human Services Survey (MBI) [19], adapted and validated to Brazilian Portuguese by Tamayo [20]. It consists of 22 questions to explore the three dimensions as follows: EE (1, 2, 3, 6, 8, 13, 14, 16, 20), DP (5, 10, 11, 15, 22) and PRP (4, 7, 9, 12, 17, 18, 19, 21). The results are scored with a five-point likert scale: “1”, never; “2”, rarely; “3”, sometimes; “4”, often; and “5”, always.

The dependent variable studied was BS and the independent variables were organized in 3 blocks (sociodemographic, lifestyle and work aspects), including sex (female = 0/male = 1); age (up to 35 years = 0/36 years or older = 1); ethnicity (not black = 0/black = 1); marital status (single = 0/married = 1); residence (urban = 0/rural = 1); having children (no = 0/yes = 1); economic situation (satisfied = 0/dissatisfied = 1); profession (nurse = 0/nursing technician = 1); satisfaction with current occupation (yes = 0/no = 1); time spent in PHC (up to 4 years = 0/5 years or more = 1); work bond (stable = 0; precarious = 1); submitted to work-related aggression (no = 0/yes = 1); night shift (no = 0/yes = 1); rest break (yes = 0/no = 1); ventilation condition (satisfactory = 0/poor = 1); temperature condition (satisfactory = 0/poor = 1); lighting condition (satisfactory = 0/poor = 1); technical resources and equipment (satisfactory = 0/precarious = 1); personal protective equipment (satisfactory = 0/precarious = 1); collective protective equipment (satisfactory = 0/precarious = 1); noise (negligible = 0/unbearable = 1); practice of physical activity (yes = 0/no = 1); smoking habit (no = 0/yes = 1); use of alcohol (no = 0/yes = 1); healthy eating (yes = 0/no = 1); satisfied with physical form (yes = 0/no = 1); last consultation with healthcare professional (less than 12 months = 0/more than 12 months = 1); quality of life (good = 0/bad = 1).

Data typing and processing were performed with the Statistical Package for Social Sciences (SPSS), version 22.0 for Windows, and data analysis on STATA for Windows version 14.0 (StataCorp, College Station, TX, USA), in the Laboratory for Teaching, Research and Extension in Collective Health (LEPESC) of the State University of Bahia (UNEB), Brazil.

Data analysis was started using descriptive statistics to characterize the sample and estimate the prevalence of the outcome, expressed as absolute and relative frequencies. The data were presented in tables and mapped by mesoregions. This procedure was done by using the software ArcGIS 10.3.

In order to analyse the Maslach Burnout Inventory, a score was obtained based on questions related to each BS dimension and with the following cut-off points: EE: high (≥27 points), moderate (19 to 26 points) and low (<19 points); DP: high (≥10 points), moderate (6 to 9 points) and low (<6 points) and RPR: high (≤33 points), moderate (34 to 39 points) and low (≥40 points) [21]. BS was dichotomized according to the criteria of Ramirez et al. [22] as present or absent when considering the existence of high scores in the dimensions of EE and DP and low scores in RPR. Cronbach’s Alpha Coefficient was applied for MBI reliability analysis.

Secondly, a bivariate analysis was performed to evaluate the gross association between independent and dependent variables, based on the calculation of prevalence ratios (PR), their respective confidence intervals (CI) at 95%, and a Pearson’s Chi-Square Test for the analysis of statistical significance based on a value of *p* ≤ 0.25 as a selection criterion for a multivariate analysis.

A two-step hierarchical analysis was conducted by using a backward logistic regression. At first, we evaluated the intrablock associations for pre-selecting the variables to the hierarchical model by using the value of *p* ≤ 0.17 as a retention criterion [23]. Next, an interblock analysis was performed with the following input hierarchy: sociodemographic (distal block), labour (intermediate block) and lifestyle (proximal block). The variables statistically associated with BS at the 5% significance level (*p* value ≤ 0.05) remained in the final model.

The PRs and their respective CIs were obtained by Poisson robust regression. This method was also used by Coutinho, Scazufca and Menezes [24] and Francisco et al. [25] to convert odds ratios (ORs) (obtained in logistic regression models) to PRs. The final model was based on Hosmer and Lemeshow’s goodness-of-fit test for adequacy analysis, the area under the Receiver Operating Characteristic (ROC) curve for data discrimination, and the Variance Inflation Factor (VIF) Statistical Test to identify possible collinearities between the variables. Then, the existence of influential observation patterns was evaluated.

The Declaration of Helsinki of the World Medical Association and Legal Resolution 466/2012 of Brazil were fully respected. The study was approved by the Research Ethics Committee involving human beings of UNEB, Brazil, opinion 872.365/2014.

## 3. Results

**Figure 1:**
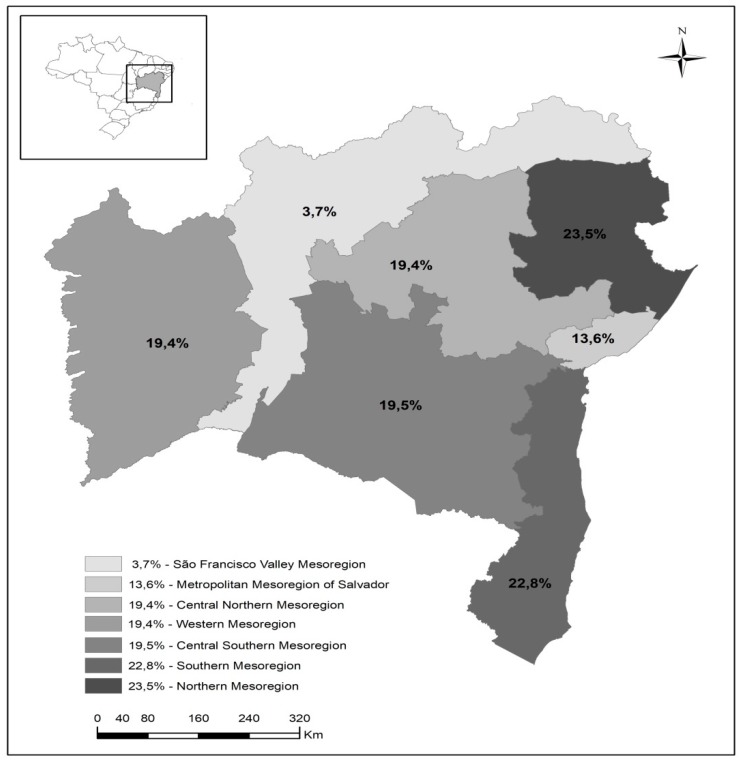
Prevalence of Burnout Syndrome in Primary Health Care nursing professionals distributed per mesoregion of Bahia, Brazil, 2018 (*n* = 1121).

Among 1125 nursing professionals evaluated, 18.3% had Burnout Syndrome, with a spatial distribution ranging from 3.7% to 23.5% (Figure 1). Most of the study population was female, black and young, with a mean age of 37.1 years (SD ± 9.6).

By assessing the levels of each MBI dimension among PHC nursing professionals, it was observed that 41.1% had a moderate level of EE, 44.5% a high level of DP and 60.2% had a high level of RPR. When analysing the means of each dimension, a moderate level of EE (22.4% ± 6.68), moderate SD (9.6 ± 3.99) and high RPR (30.4 ± 6.27) were observed. The internal reliability of MBI dimensions was evaluated by Cronbach’s Alpha Coefficient, obtaining values >0.70; i.e., characterized as reliable and with good internal consistency. The reliability coefficient in EE was 0.82, in SD was 0.79 and RPR was 0.81 (Table 1).

In the bivariate analysis, the variables of the distal block (Block 1) female sex (PR = 0.52; CI 95% = 0.39–0.69), race/black (PR = 0.64; CI 95% = 0.49–0.84), rural residence (PR = 2.17; CI 95% = 1.68–2.79) and dissatisfaction with economic situation (PR = 1.40; CI 95% = 1.09–1.81) were associated with BS. There was a higher prevalence of the outcome among nursing professionals who were over 36 years old, married, rural residents, with children and dissatisfied with the economic situation. In the proximal block (Block 3), the variables of physical inactivity (PR = 1.74; CI 95% = 1.35–2.23), smoking (PR = 2.27; CI 95% = 1.73–2.96) and dissatisfaction with their physical form (PR = 1.71; CI 95% = 1.32–2.21) were associated with the syndrome. A higher prevalence of BS was observed in professionals with the following habits: without a physical activity routine, smoking habits, alcohol consumption, without healthy eating, who were dissatisfied with their physical form, who went more than 12 months without consulting with health professionals, and without an appropriate quality of life (Table 2).

Among the variables of the intermediate block (Block 2), dissatisfaction with current occupation (PR = 2.35; CI 95% = 1.81–3.06), time spent in PHC of greater than 5 years (PR = 1.42; CI 95% = 1.10–1.83), absence of rest breaks (PR = 1.48; CI 95% = 1.15–1.89), submitted to work-related aggression (PR = 1.98; CI 95% = 1.55–2.52) and night shifts (PR = 2.00; CI 95% = 1.55–2.56) were associated with BS. There was a higher prevalence of outcomes between nursing technicians, dissatisfaction with current occupation, absence of rest breaks, being submitted to work-related aggression, night shift and poor ventilation, temperature, lighting and technical resources and equipment (Table 3).

The following were statistically associated with BS in the logistic regression: ethnicity (PR = 062; CI 95% = 0.47–0.83), place of residence (PR = 2.35; CI 95% = 1.79–3.09), economic situation (PR = 1.40; CI 95% = 1.06–1.86), satisfaction with current occupation (PR = 1.75; CI 95% = 1.31–2.33), being submitted to work-related aggression (PR = 1.60; CI 95% = 1.23–2.08), rest break (PR = 1.83; CI 95% = 1.41–2.37), technical resources and equipment (PR = 1, 37; CI 95% = 1.06–1.77), night shift (PR = 1.49; CI 95% = 1.14–1.96), physical activity routine (PR = 1.72; CI 95% = 1 28–2.31), smoking (PR = 1.82; CI 95% = 1.35–2.45), and satisfaction with physical form (PR = 1.34; CI 95% = 1.01–1.79). It is noteworthy that the final model exhibited an ROC curve of 0.80 and Hosmer and Lemeshow’s goodness-of-fit test score of 0.81, indicating an adequate discriminatory power and appropriate adjustment to the data. No collinearity was found between the variables (VIF < 5).

Influential observation patterns were identified. When such patterns were excluded, the final model was better adjusted and increased from 0.21 to 0.81; the discriminatory power from 0.77 to 0.80 and the Maximum Likelihood Test showed statistical differences between the complete (all observations) and the reduced (without influential data) model. Thus, influential data were excluded in the final model (Table 4).

## 4. Discussion

This is an unprecedented investigation in the state of Bahia, Brazil which was focused on the factors associated with BS in PHC nursing professionals. There are relatively few studies that adopted models that jointly evaluated social, economic, labour and lifestyle factors associated with BS among PHC nursing professionals, although some studies have proposed other conditions of mental illness in the PHC nursing setting [26,27].

After using the multivariate analysis with hierarchical block entry, the following factors were most relevant to the occurrence of BS: ethnicity, residence in rural areas, dissatisfaction with the economic situation and current occupation, being submitted to work-related aggression, not having a break for rest, poor technical resources and equipment, night shift routine, absence of physical activity routine, smoking, and dissatisfaction with physical form.

In the block of sociodemographic variables, when comparing the groups regarding ethnicity, there was a higher probability of BS among non-black people. Protective factors from resilience may support such findings. According to Cyrulnik [28], resilience implies not only talking about the risks posed by the circumstances experienced by the subject, which increase the probability of problems or maladaptation, but also recognizing the concomitant existence of certain conditions or processes that would protect the subjects. Living in rural areas increased the occurrence of BS two-fold. No mechanism was found in the literature to explain this finding. Because it is a young population, the lack of leisure activity and physical activity program, lack of professional advancement, precariousness of work in rural PHC, and long-distance travel are the explanatory factors of this event.

Satisfaction with compensation has been one of the factors that influence the quality of life of nursing professionals [29]. In this sense, the dissatisfaction with the economic situation observed in this study may be justified by the socioeconomic difficulties experienced by the working class, given that nursing is still somewhat socially undervalued, which implies, among other things, unsatisfactory compensation [30]. Thus, there is a search for other work bonds, leading to greater occupational stress [31]; that is, a greater likelihood of suffering from BS.

Regarding the labour block, dissatisfaction with the current occupation, aggression in the work environment, absence of rest breaks, precarious technical resources and equipment, and night shifts were associated with BS. All variables mentioned are related to BS, according to the literature [32,33,34,35,36]. There is a paradox about the ideals and the reality of PHC in Brazil. PHC should function as a priority for the Brazilian Public Health System (SUS—*Sistema Único de Saúde*) with prevention, health promotion and comprehensive care as its scope [37]. The reality is based on actions focused on the disease, fragmented work, high demand from patients/family/community, often conflicting interpersonal relationships, low wages, situations of intense suffering, and emphasis on situations of violence that are common in Brazil. These demeaning conditions predict occupational stress and BS among nursing professionals [9,38,39,40].

The lifestyle block indicates that not performing physical activity, smoking, and dissatisfaction with physical form were associated with BS. It is likely that harmful lifestyle habits are related to a low level of the quality of life of the individual, bringing impacts on the work environment and, therefore, increasing occupational stress [41] and BS. A quasi-experimental clinical study conducted in China found that a physical exercise program established a better workplace health behaviour and relieved BS [42]. Thus, physical activity reduces stress, provides psychological balance and stimulates social interaction [43], and thus is a protective factor for BS.

This study revealed a high prevalence of BS among PHC nursing professionals, corresponding to 18.3%, with a spatial distribution ranging from 3.7% to 23.5%. Distinct results on the prevalence of BS were found among investigations involving nursing professionals from various work sectors, especially those conducted by Rossi, Santos and Passos [44], Holmes et al. [45], Falgueras et al. [46], Ferreira and Lucca [47], Merces et al. [48], Lima et al. [49], Chico-Barba et al. [10], with BS prevalences of 10%, 11.1%, 9.6%, 5.9%, 58.3%, 56.6%, and 19.6%, respectively. However, the variation in prevalence in the studies cited is justified by the multiple criteria used to evaluate BS. In this study, the criterion chosen is the most widespread in the national and international literature, as it does not overestimate BS [9,10,22,47].

When analysing the distribution of the mean points of the BS dimensions, there was a higher frequency of EE is at moderate level, the DP and RPR were at high levels, and the RPR had a higher frequency. A study conducted with PHC nurses in Burgos, Spain indicated a high degree in the three dimensions, and RPR was also more frequent, which confirms our findings [50]. The number of models which aim to explain the path of BS is vast. While some authors believe that DP is the trigger of the process, others argue that the EE dimension is strongly associated to the development of DP and RPR [19,51,52].

These lines of thinking are believed to be obsolete. If BS were only associated to EE or DP, the term “burnout” would be unnecessary, as these dimensions by themselves do not represent the whole “story” of BS [7]. If a nursing professional has autonomy, access to necessary technical resources and equipment and experiences as well as a mental load reduction, work engagement will be inevitable. However, when recognition and reward are insufficient, whether in financial, institutional or social terms, there will be increased vulnerability and a cascade effect of reactions leading to RPR, DP, EE and, consequently, BS, regardless of the order [4,7].

The robust method used in this study confirmed the predictive ability of the results; however, some limitations should be noted: (i) a cross-sectional study cannot establish a causal relationship; (ii) there is a susceptibility to the occurrence of memory bias due to self-reported variables and healthy worker effect. Given the limitations described, some methodological precautions were taken: (i) the 94.1% response rate was acceptable for population-based studies [53]; (ii) the hierarchical model provided good guidance for multivariate analysis, contributing to the creation of the final model and supporting the interpretation of the results in light of the theoretical, social and health aspects.

## 5. Conclusions

The prevalence of BS among PHC nursing professionals is high. The RPR dimension presented a high frequency. The variables associated with the syndrome were ethnicity, place of residence, economic status, current occupation, aggression at work, rest breaks, poor technical resources and equipment, night shift, physical activity, smoking, and dissatisfaction with physical form.

Strategies are needed to prevent BS at intra and extra-organizational levels, with emphasis on the implementation of workers’ health programs in PHC. Such initiatives should stimulate lifestyle changes, improve working conditions, focus on new technologies, and improve health and the environment, as well as providing social support at work. Studies on BS among PHC nursing professionals should be increased since there is a shortage of investigations with robust methods.

## Figures and Tables

**Table 1:** Frequency distribution of the Emotional Exhaustion (EE), Depersonalization (DP) and Reduced Professional Achievement (RPR) dimensions among Primary Health Care nursing professionals in Bahia, Brazil in 2018 (*n* = 1121).

Dimensions	*n* (%)			Average Points	Standard Deviation	Cronbach’s Alpha
	Low	Moderate	High			
EE	346 (30.8)	461 (41.1)	316 (28.1)	22.4	6.68	0.82
DP	220 (19.6)	403 (35.9)	501 (44.5)	9.6	3.99	0.79
RPR	40 (3.6)	407 (36.2)	677 (60.2)	30.4	6.27	0.81

**Table 2:** Gross prevalence ratio of Burnout Syndrome and its 95% confidence intervals, according to sociodemographic and lifestyle variables in Primary Health Care nursing professionals in Bahia, Brazil in 2018 (*n* = 1121).

		Burnout Syndrome		
Variables	*n* (%)	P (%) ^b^	PR ^c^ (CI 95%) ^d^	*p*-Value ^e^
Sociodemographic (Distal Block)				
Sex (*n* = 1125)				
Male	136 (12.1)	43 (31.6)	1.00	
Female	989 (87.9)	162 (16.4)	0.52 (0.39–0.69)	<0.01 *
Age (*n* = 1125)				
Up to 35 years old	587 (52.2)	101 (17.3)	1.00	
36 years old or older	538 (47.8)	104 (19.4)	1.12 (0.87–1.43)	0.36
Ethnicity (*n* = 1098) ^a^				
Non-black people	246 (22.4)	61 (24.8)	1.00	
Black people	852 (77.6)	136 (16.0)	0.64 (0.49–0.84)	<0.01 *
Marital Ssatus (*n* = 1125)				
Not married	606 (53.9)	106 (17.6)	1.00	
Married	519 (46.1)	99 (19.1)	1.08 (0.84–1.39)	0.50
Place of residence (*n* = 1125)				
Urban	941 (83.6)	144 (15.4)	1.00	
Rural	184 (16.4)	61 (33.3)	2.17 (1.68–2.79)	<0.01 *
Children (*n* = 1125)				
No	454 (40.4)	89 (16.7)	1.00	
Yes	671 (59.6)	116 (17.3)	0.87 (0.68–1.12)	0.30
Economic situation (*n* = 1125)				
Satisfied	573 (50.9)	87 (15.2)	1.00	
Dissatisfied	552 (49.1)	118 (21.5)	1.40 (1.09–1.81)	<0.01 *
Lifestyle (proximal block)				
Routine of physical activities (*n* = 1125)				
Yes	639 (56.8)	88 (13.8)	1.00	
No	486 (43.2)	117 (24.1)	1.74 (1.35–2.23)	<0.01 *
Smoking habit (*n* = 1125)				
No	992 (88.2)	157 (15.9)	1.00	
Yes	133 (11.8)	48 (36.1)	2.27 (1.73–2.96)	<0.01 *
Alcohol consumption (*n* = 1125)				
No	1083 (96.3)	194 (18.0)	1.00	
Yes	42 (3.7)	11 (26.2)	1.45 (0.86–2.45)	0.18
Healthy eating (*n* = 1125)				
Yes	590 (52.4)	96 (16.3)	1.00	
No	535 (47.6)	109 (20.5)	1.25 (0.98–1.61)	0.07
Satisfaction with physical form (*n* = 1125)				
Yes	571 (50.8)	77 (13.5)	1.00	
No	554 (49.2)	128 (23.2)	1.71 (1.32–2.21)	<0.01 *
Last consultation with health professional (*n* = 1125)				
Less than 12 months	924 (82.1)	164 (17.8)	1.00	
More than 12 months	201 (17.9)	41 (20.4)	1.14 (0.84–1.55)	0.40
Quality of life (*n* = 1125)				
Good	836 (74.3)	147 (17.6)	1.00	
Poor	289 (25.7)	58 (20.1)	1.14 (0.86–1.49)	0.35

^a^ Variable with missing data; ^b^ P: prevalence of outcome between exposed and unexposed; ^c^ PR: gross prevalence ratio; ^d^ CI 95%: confidence Iitervals of 95%; ^e^ Chi test—Pearson’s square; * statistical significance.

**Table 3:** Gross prevalence ratio of Burnout Syndrome and its 95% confidence intervals, according to labour variables in Primary Health Care nursing professionals in Bahia, Brazil in 2018 (*n* = 1121).

		Burnout Syndrome		
Variables	*n* (%)	P (%) ^a^	PR ^b^ (CI 95%) ^c^	*p*-Value ^d^
Labour (intermediate block)				
Profession (*n* = 1125)				
Nurse	455 (40.4)	80 (17.8)	1.00	
Nursing technician	670 (59.6)	125 (18.7)	1.05 (081–1.35)	0.70
Satisfaction with current occupation (*n* = 1125)				
Yes	987 (87.7)	154 (15.7)	1.00	
No	138 (12.3)	51 (37.0)	2.35 (1.81–3.06)	<0.01 *
Occupation time in PHC (*n* = 1125)				
Up to 4 years	555 (49.3)	83 (15.0)	1.00	
5 years or more	570 (50.7)	122 (21.4)	1.42 (1.10–1.83)	<0.01 *
Work bond (*n* = 1125)				
Stable	866 (77.0)	159 (18.4)	1.00	
Precarious	259 (23.0)	46 (18.0)	0.97 (0.72–1.31)	0.88
Rest break (*n* = 1125)				
Yes	672 (59.7)	103 (15.3)	1.00	
No	453 (40.3)	102 (22.7)	1.48 (1.15–1.89)	<0.01 *
Submitted to work-related aggression (*n* = 1125)				
No	751 (66.8)	103 (13.8)	1.00	
Yes	374 (33.2)	102 (27.3)	1.98 (1.55–2.52)	<0.01 *
Night shift (*n* = 1125)				
No	894 (79.5)	135 (15.2)	1.00	
Yes	231 (20.5)	70 (30.3)	2.00 (1.55–2.56)	<0.01 *
Ventilation condition (*n* = 1125)				
Satisfactory	662 (58.8)	115 (17.5)	1.00	
Poor	463 (41.2)	90 (19.4)	1.11 (0.87–1.43)	0.40
Temperature condition (*n* = 1125)				
Satisfactory	602 (53.5)	106 (17.6)	1.00	
Poor	523 (46.5)	99 (19.0)	1.08 (0.84–1.38)	<0.01 *
Lighting condition (*n* = 1125)				
Satisfactory	1038 (92.3)	185 (17.9)	1.00	
Poor	87 (7.7)	20 (23.3)	1.30 (0.87–1.95)	0.21
Technical resources & equipment (*n* = 1125)				
Satisfactory	543 (48.3)	87 (16.1)	1.00	
Poor	582 (51.7)	118 (20.3)	1.27 (0.98–1.63)	0.06
Personal protective equipment (*n* = 1125)				
Satisfactory	599 (53.2)	112 (18.8)	1.00	
Poor	526 (46.8)	93 (17.7)	0.95 (0.74–1.21)	0.66
Collective protective equipment (*n* = 1125)				
Satisfactory	509 (45.2)	100 (19.7)	1.00	
Poor	616 (54.8)	105 (17.1)	0.87 (0.68–1.11)	0.27
Noise (*n* = 1125)				
Negligible	608 (54.0)	113 (18.7)	1.00	
Unbearable	517 (46.0)	92 (17.8)	0.95 (0.74–1.22)	0.71

^a^ P: prevalence of outcome between exposed and unexposed; ^b^ PR: gross prevalence ratio; ^c^ CI 95%: 95% confidence intervals; ^d^ chi test—Pearson’s square; *statistical significance. PHC: Primary Health Care.

**Table 4:** Factors associated with Metabolic Syndrome in Primary Health Care nursing professionals, obtained by multivariate analysis.

Factors Associated with Burnout Syndrome	PR_adjusted_	CI (95%)
Ethnicity	0.62	(0.47–0.83)
Place of residence	2.35	(1.79–3.09)
Economic situation	1.40	(1.06–1.86)
Satisfaction with current occupation	1.75	(1.31–2.33)
Submitted to work-related aggression	1.60	(1.23–2.08)
Rest break	1.83	(1.41–2.37)
Technical Resources and Equipment	1.37	(1.06–1.77)
Night shift	1.49	(1.14–1.96)
Physical activity practice	1.72	(1.28–2.31)
Smoking habit	1.82	(1.35–2.45)
Satisfaction with physical form	1.34	(1.01–1.79)
Area under the ROC curve	0.80	
Goodness-of-fit test ^¥^	0.81	

^¥^ Hosmer-Lemershow. ROC: receiver operating characteristic.

## References

[1] Antunes R.A., Ferreira L.C. (1997). Centralidade do trabalho hoje. A Sociologia No Horizonte do Século XXI.

[2] Ceolin G.F. (2014). Crise do capital, precarização do trabalho e impactos no Serviço Social. Serv. Soc. Soc..

[3] Antunes R. (2015). Adeus ao trabalho?. Ensaio Sobre as Metamorfoses e A Centralidade no Mundo do Trabalho.

[4] Dejours C. (2015). A Loucura do Trabalho: Estudo de Psicopatologia do Trabalho.

[5] Merces M.C., Silva D.S., Lopes R.A., Lua I., Silva J.K., Oliveira D.S., Servo M.L.S. (2015). Síndrome de Burnout em enfermeiras da atenção básica à saúde: Uma revisão integrativa. Rev. Epidemiol. Control. Infect..

[6] Cooper C.L., Dewe P.J., O’Driscoll M.P. (2001). Estresse Organizacional. Uma Revisão e Crítica da Teoria.

[7] Maslach C., Leiter M. (2016). Understanding the burnout experience: Recent research and its implications for psychiatry. World Psychiatry.

[8] Willard-Grace R., Hessler D., Rogers E., Dubé K., Bodenheimer T., Grumbach K. (2014). Team structure and culture are associated with lower burnout in primary care. J. Am. Board Fam. Med..

[9] Merces M.C., Souza D.S.S., Lua I., Oliveira D.S., Souza M.C. (2016). Burnout syndrome and abdominal adiposity among Primary Health Care nursing professionals. Psicol. Reflex Crit..

[10] Chico-Barba G., Jiménez-Limas K., Sánchez-Jiménez B., Sámano R., Rodríguez-Ventura A.L., Castillo-Pérez R., Tolentino M. (2019). Burnout and Metabolic Syndrome in Female Nurses, An Observational Study. Int. J. Environ. Res. Public Health.

[11] World Health Organization (2019). International Classification of Diseases 11th Revision (ICD-11). The Global Standard for Diagnostic Health Information.

[12] Maslach C., Schaufeli W.B., Leiter M.P. (2001). Job burnout. Ann. Rev. Psychol..

[13] Cañadas-De la Fuente G.A., Vargas C., San Luis C., García I., Cañadas G.R., De la Fuente E.I. (2015). Risk factors and prevalence of burnout syndrome in the nursing profession. Int. J. Nurs. Stud..

[14] Nascimento Sobrinho C.L., Barros D.S., Tironi M.O.S., Marques Filho E.S. (2010). Médicos de UTI, prevalência da Síndrome de Burnout, características sociodemográficas e condições de trabalho. Rev. Bras. Educ. Med..

[15] Gómez-Urquiza J.L., Vargas C., De la Fuente E.I., Fernández-Castillo R., Canadas-De la Fuente G.A. (2017). Age as a risk factor for burnout syndrome in nursing professionals: A meta-analytic study. Res. Nurs. Health.

[16] Vasconcelos E.M., De Martino M.M.F. (2017). Preditores da síndrome de burnout em enfermeiros de unidade de terapia intensiva. Rev. Gaúcha Enferm..

[17] Instituto Brasileiro de Geografia e Estatística (IBGE) (1990). Divisão Regional do Brasil em Mesorregiões e Microrregiões Geográficas.

[18] Seigel D.G., Podgor M.J., Remaley N.A. (1992). Acceptable values of kappa for comparison of two groups. Am. J. Epidemiol..

[19] Maslach C., Jackson S.E. (1986). Maslach Burnout Inventory.

[20] Tamayo M.R. (1997). Relation between Burnout Syndrome and Organizational Values inthe Nursing Staff of Two Public Hospitals. Master’s Thesis.

[21] Moreira D.S., Magnago R.F., Sakae T.M., Magajewski F.R. (2009). Prevalência da Síndrome de Burnout em trabalhadores de enfermagem de um hospital de grande porte da Região Sul do Brasil. Cad. De Saúde Pública.

[22] Ramirez A.J., Graham J., Richards M.A., Cull A., Gregory W.M. (1996). Mental health of hospital consultants: The effects of stress and satisfaction at work. Lancet.

[23] Hosmer D.W., Lemeshow S. (2000). Applied Logistic Regression.

[24] Coutinho L.M.S., Scazufca M., Menezes P.R. (2008). Methods for estimating prevalence ratios in crosssectional studies. Rev. Saúde Pública.

[25] Francisco P., Donalisio M., Barros M., Cesar C., Carandina L., Goldbaum M. (2008). Association measures in cross-sectional studies with complex samplings: Odds ratio and prevalence ratio. Rev. Bras. Epidemiol..

[26] Lua I., Araújo T.M., Santos K.O.B., Almeida M.M.G. (2018). Factors associated with common mental disorders among female nursing professionals in primary health care. Psicol. Reflexão E Crítica.

[27] Lua I., Almeida M.M.G., Araújo T.M., Soares J.F.S., Santos K.O.B. (2018). Autoavaliação negativa da saúde em trabalhadoras de enfermagem da atenção básica. Trab. Educ. Saúde..

[28] Cyrulnik B. (2001). Les Vilains Petits Canards.

[29] Amaral J.F., Ribeiro J.P., Paixão D.X. (2015). Qualidade de vida no trabalho dos profissionais de enfermagem em ambiente hospitalar: Uma revisão integrativa. Rev. Espaço. Saúde..

[30] Secco I.A.O., Robazzi M.L.C.C., Souza F.E.A., Shimizu D.S. (2010). Cargas psíquicas de trabalho e desgaste dos trabalhadores de enfermagem de hospital de ensino do Paraná, Brasil. Rev. Elet. Saúde Ment. Álcool. Drog..

[31] Merces M.C., Cordeiro T.M.S.C., Santana A.I.C., Lua I., Silva D.S., Alves M.S., D’Oliveira Júnior A. (2016). Síndrome de burnout em trabalhadores de Enfermagem da Atenção Básica à Saúde. Rev. Baiana De Enferm..

[32] Kalliath T., Morris R. (2002). Job satisfaction among nurses, A predictor of burnout levels. J. Nurs. Adm..

[33] Lorenz V.R., Guirardello E.B. (2014). The environment of professional practice and Burnout in nurses in primary healthcare. Rev. Lat. Am. Enferm..

[34] Khamisa N., Oldenburg B., Peltzer K., Ilic D. (2015). Work Related Stress, Burnout, Job Satisfaction and General Health of Nurses. Int. J. Environ. Res. Public Health.

[35] Alameddine M., Mourad Y., Dimassi H. (2015). A national study on nurses’ exposure to occupational violence in Lebanon: Prevalence, consequences and associated factors. PLoS ONE.

[36] Vidotti V., Ribeiro R.P., Galdino M.J.Q., Martins J.T. (2018). Burnout Syndrome and shift work among the nursing staff. Rev. Latino. Am. Enfermagem..

[37] Starfield B. (2002). Atenção Primária: Equilíbrio Entre Necessidades de Saúde, Serviços e Tecnologias.

[38] McHugh M.D., Stimpfel A.W. (2012). Nurse reported quality of care: A measure of hospital quality. Res. Nurs. Health.

[39] Yao Y., Yao W., Wang W., Li H., Lan Y. (2013). Investigation of risk factors of psychological acceptance and Burnout syndrome among nurses in China. Int. J. Nurs. Pract..

[40] Merces M.C., Gomes A.M.T., Guimarães E.L.P., Santana A.I.C., Silva D.S., Machado Y.Y., Couto P.L.S., França L.C.M., Figueiredo V.P., D’Oliveira Júnior A. (2018). Burnout and metabolic conditions are professionals of nursing, A pilot study. Enferm. Bras..

[41] Zanelli J.C. (2010). Estresse Nas Organizações de Trabalho. Compreesão e Intervenção Baseadas em Evidências.

[42] Tsai H.H., Yeh C.Y., Su C.T., Chen C.J., Peng S.M., Chen R.Y. (2013). The effects of exercise program on burnout and metabolic syndrome components in banking and insurance workers. Ind. Health.

[43] Masters K.S., Lacaille R.A., Shearer D.S. (2003). The acute affective response of Type A behavior pattern individuals to competitive and noncompetitive exercise. Can. J. Behav. Sci..

[44] Rossi S.S., Santos P.G., Passos J.P. (2010). A síndrome de burnout no enfermeiro: Um estudo comparativo entre atenção básica e setores fechados hospitalares. J. Res. Fundam. Care.

[45] Holmes E.S., Santos S.R., Farias J.Á., Costa M.B.S. (2014). Síndrome de burnout em enfermeiros na atenção básica: Repercussão na qualidade de vida. Can. J. Behav. Sci..

[46] Falgueras M.V., Munoz C.C., Pernas F.O., Sureda J.C., López M.P.G., Miralles J.D. (2015). Burnout y trabajo en equipo en los profesionales de Atención Primaria. Aten Primaria.

[47] Ferreira N.N., Lucca S.R. (2015). Burnout syndrome in nursing assistants of a public hospital in the state of São Paulo. Rev. Bras. Epidemio..

[48] Merces M.C., Lopes R.A., Silva D.S., Oliveira D.S., Lua I., Mattos A.I., D’Oliveira Júnior A. (2017). Prevalência da Síndrome de Burnout em profissionais de enfermagem da atenção básica à saúde. Rev. Fund. Care Online.

[49] Lima A., Farah B.F., Bustamante–Teixeira M.T. (2018). Análise da prevalência da Síndrome de Burnout em profissionais da atenção primária em saúde. Trab. Educ. Sáude.

[50] Cámara R.S., Cuesta M.I.S. (2005). Prevalence of burnout in primary care nurses. Enfermería Clínica.

[51] Golembiewski R.T., Munzenrider R., Carter D. (1983). Phases of progressive burnout and their work site covariants, Critical issues in OD research and praxis. J. Appl. Behav. Sci..

[52] Leiter M.P., Schaufeli W.B., Maslach C., Marek T. (1993). Burnout as a development process, considerations of models. Professional Burnout: Recents Developments in Theory and Research.

[53] Rodriguez-Artalejo F., Graciani A., Guallar-Castillon P., León-Munoz L.M., Zuluaga M.C., López-Garcia E., Gutierrez-Fisac J.L., Taboada J.M., Aguilera M.T., Regidor E. (2011). Rationale and methods of the study on nutrition and cardiovascular risk in Spain (ENRICA). Rev. Esp. Cardiol..

